# A nonporous crystalline organic cage for selective water uptake and storage

**DOI:** 10.1039/d5sc06328k

**Published:** 2025-11-10

**Authors:** Lukman O. Alimi, Nida Khalfay, Soumaya Khlifi, Weibin Lin, Basem Moosa, Niveen M. Khashab

**Affiliations:** a Smart Hybrid Materials Laboratory (SHMs), Physical Science and Engineering, King Abdullah University of Science and Technology (KAUST) Thuwal 23955-6900 Saudi Arabia niveen.khashab@kaust.edu.sa

## Abstract

A nonporous crystalline organic cage (Oba-cage) effectively captures and stores water from a mixed organic solvent/water system. The observed water uptake capacities of 106.2 mg g^−1^ (10.6 wt%) at 0 °C and 102.1 mg g^−1^ (10.2 wt%) at 25 °C are significant for nonporous cages, as collectively confirmed by different techniques. This excellent water uptake and storage performance is attributed to the strong host–guest O–H⋯N hydrogen bonding interactions between the imine nitrogen atoms of the cage and water molecules. These findings establish organic cages as a promising class of compounds for water harvesting and storage applications.

## Abstract 2

A nonporous crystalline organic cage (Oba-cage) effectively adsorbs water from mixed solvent systems, showing high capacities of 106.2 mg g^−1^ (0 °C) and 102.1 mg g^−1^ (25 °C), highlighting its potential as an efficient water storage material.

## Graphical abstract

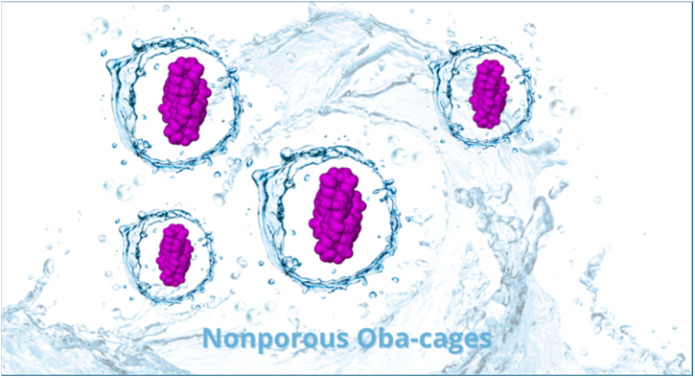

## Introduction

The development of multi-functional, cost-effective, and environmentally sustainable materials is essential for meeting the ever-growing demand for smart materials that can selectively store water. These demands continue to drive research to discover, prepare and investigate new materials capable of efficient water capture, storage, and purification. Traditional porous materials, such as metal–organic frameworks (MOFs) and covalent organic frameworks (COFs), have dominated this field due to their high surface areas and tunable porosity.^1–3^ However, their susceptibility to hydrolysis, structural collapse in aqueous environments, and energy-intensive regeneration processes limit their practicality.^4,5^ Recent advances in self-assembled organic cages with defined cavities offer a promising alternative.^6–13^ Among these, nonporous adaptive crystals emerge as innovative candidates for water storage, combining structural stability, design flexibility, and unique host–guest interaction mechanisms.^8–11,13,14^

Organic cages, such as porous organic cages (POCs), are celebrated for their modular synthesis, solution processability, and customizable functionality.^6,7^ For instance, hydrazone-linked POCs exhibit exceptional water stability and pollutant removal efficiency, underscoring their potential in aqueous applications.^12^ However, the focus on nonporous crystalline cages introduces a paradigm shift although not extensively explored yet for water related applications. Unlike their porous counterparts, these materials lack interconnected voids but leverage crystalline order and tailored molecular cavities to interact with guest molecules such as water.^8–11,13,14^ For example, imine-based cages like CC3 demonstrate reversible water uptake (20.1 wt%) despite their porous nature,^15^ suggesting that nonporous analogues could achieve similar efficiency through surface interactions or extrinsic cavities.

The flexible conformation of nonporous crystalline cages is also very important in achieving such efficient guest molecule uptake and storage.^9,13,14^ By modifying functional groups or linker geometries, their affinity for guest molecules can be optimized. Recent work on DIHO-cage reveals how substituent type or size (*e.g.*, hydroxyl group) influences the guest adsorption properties.^8^ Also, the Oba-cage through its flexible C–O single bond could influence the selective sorting of the monohalotoluene isomers based on the size of the guest substituent halogens.^9^ We believe that these structural adjustments could also enhance water adsorption kinetics or capacity. Apart from structural flexibility, nonporous crystalline cages also address stability concerns. For example, the amorphous nonporous superphane cages exhibit record-breaking iodine adsorption in water,^16^ proving that nonporous materials, either amorphous or crystalline, can outperform porous ones in hydrophilic environments.

**Fig. 1:**
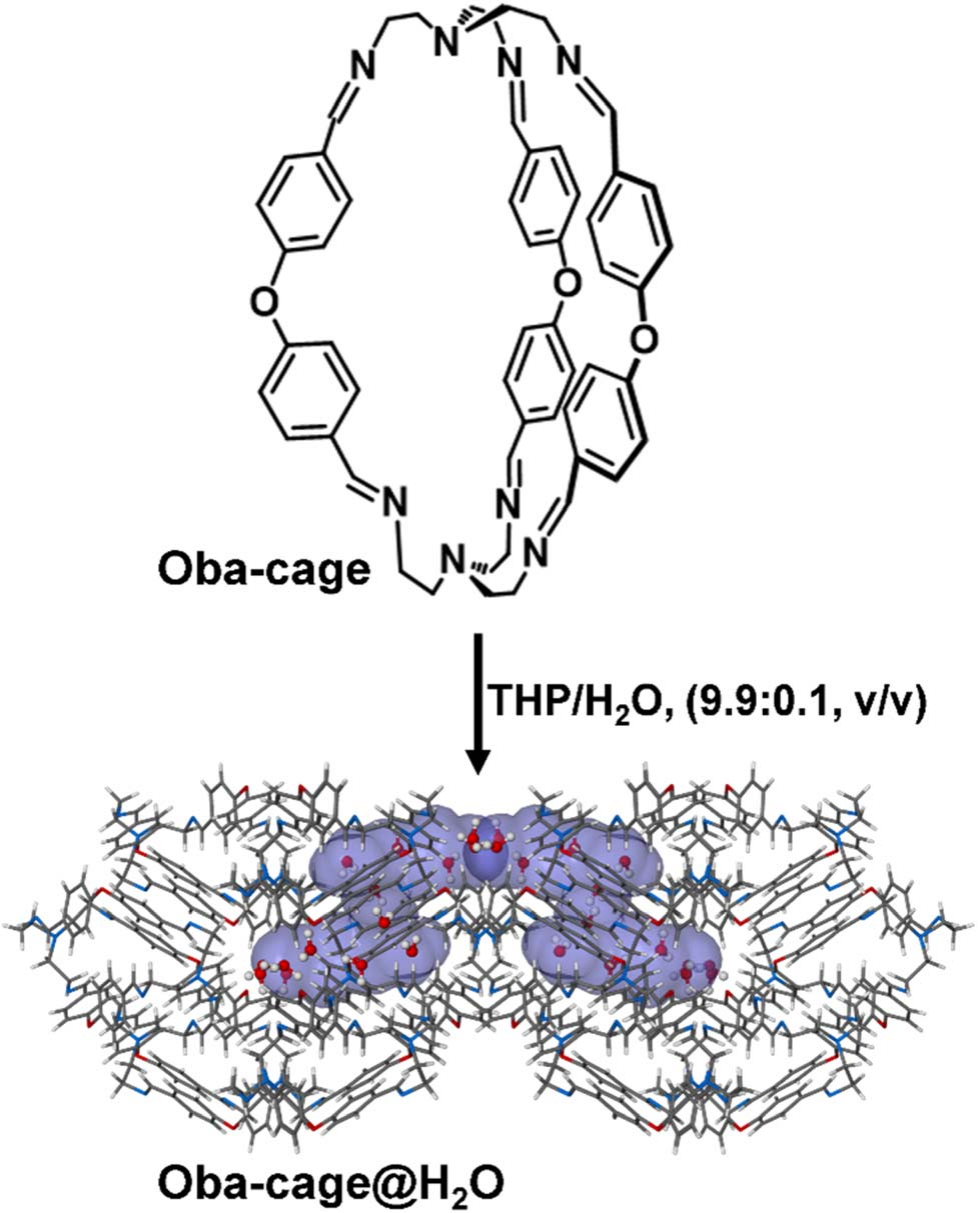
Representation of the Oba-cage as an adsorptive material for selective water uptake and storage.

Presently, there are no examples of nonporous crystalline organic cages with high water uptake and storage.^17^ Most available examples reported the use of porous crystalline/amorphous organic cages or salts for water uptake only.^14,15^ Thus, tunable nonporous crystalline organic cages could present a transformative approach to water capture and storage because of their unique structural and functional qualities, unlike other porous materials with permanent or fixed pore size and dimensions that as a result can only adsorb or separate guest molecules based on their size and/or shape.^1–3,18,19^ These nonporous organic cages exhibit guest-induced porosity which further allows for dynamic and highly selective uptake of some guest molecules like water, while remaining inaccessible to other small molecules under similar conditions strictly due to host–guest interactions.^8–11^ By combining precise structural control with robust crystalline architecture, these materials could bypass the limitations of traditional porous systems if carefully harnessed. Herein, we utilized the Oba-cage (Fig. 1), a highly flexible imine-based organic cage, as an adsorptive material for selective water capture and storage. While the Oba-cage has demonstrated the ability to selectively adsorb *ortho*-isomers of halotoluenes from mixtures by leveraging host–guest interactions and structural adaptability in various solvents, there was no evidence showing its application for water uptake or storage (Fig. 1). Furthermore, the water adsorption capacities of 106.2 mg g^−1^ (10.6 wt%) at 0 °C and 102.1 mg g^−1^ (10.2 wt%) at 25 °C achieved by the Oba-cage are, to the best of our knowledge, the highest reported for nonporous crystalline materials. This represents the first demonstration of a nonporous crystalline organic cage functioning as an efficient material for selective water capture.

## Results and discussion

The Oba-cage was synthesized as earlier reported through a simple, cost effective and one-step condensation reaction of 4,4′-oxybis(benzaldehyde) with flexible tris(2-aminoethyl) amine (TREN) in acetonitrile with good 82% yield (Scheme S1).^20^ Proton, ^13^C NMR, FT-IR and mass spectra confirmed the successful synthesis of the Oba-cage (Fig. S1–S3). We believe that the Oba-cage could further be explored because of its flexible conformation and ability to form host–guest complexes which are essential for water uptake and storage studies.^9^ These two features have earlier been demonstrated by the Oba-cage in fabricating a vapor responsive composite film and the selective sorting of monohalotoluene isomers.^9,20^ In the same reports we have demonstrated the high tendency of the Oba-cage to form host–guest complexes with most organic solvents.^9^ For example, we reported two different single crystals Oba-cage@oCT and Oba-cage@CHCl_3_ obtained from two separate chlorinated solvents namely CHCl_3_ (Oba-cage-CHCl_3_) and *ortho*-chlorotoluene (Oba-cage-oCT) (Fig. S4 and Table S1). The solvent molecules in these two crystal structures are trapped in the channels of the crystal packing forming host/guest complexes (Fig. S5).^9,20^

Thermogravimetric analysis (TGA) of the already activated Oba-cage shows no appreciable weight loss below 300 °C indicating that the material contains no residual solvent after activation at 60 °C for 6 h (Fig. S6). Powder X-ray diffraction (PXRD) patterns confirm the bulk purity of the as-synthesized Oba-cage and that the activated Oba-cage also retains its crystallinity after desolvation (Fig. S7). The Brunauer–Emmett–Teller (BET) surface area of the activated Oba-cage was determined to be 23 m^2^ g^−1^ by using a N_2_ gas sorption isotherm at 77 K, indicating that the activated Oba-cage is nonporous (Fig. S8).

**Fig. 2:**
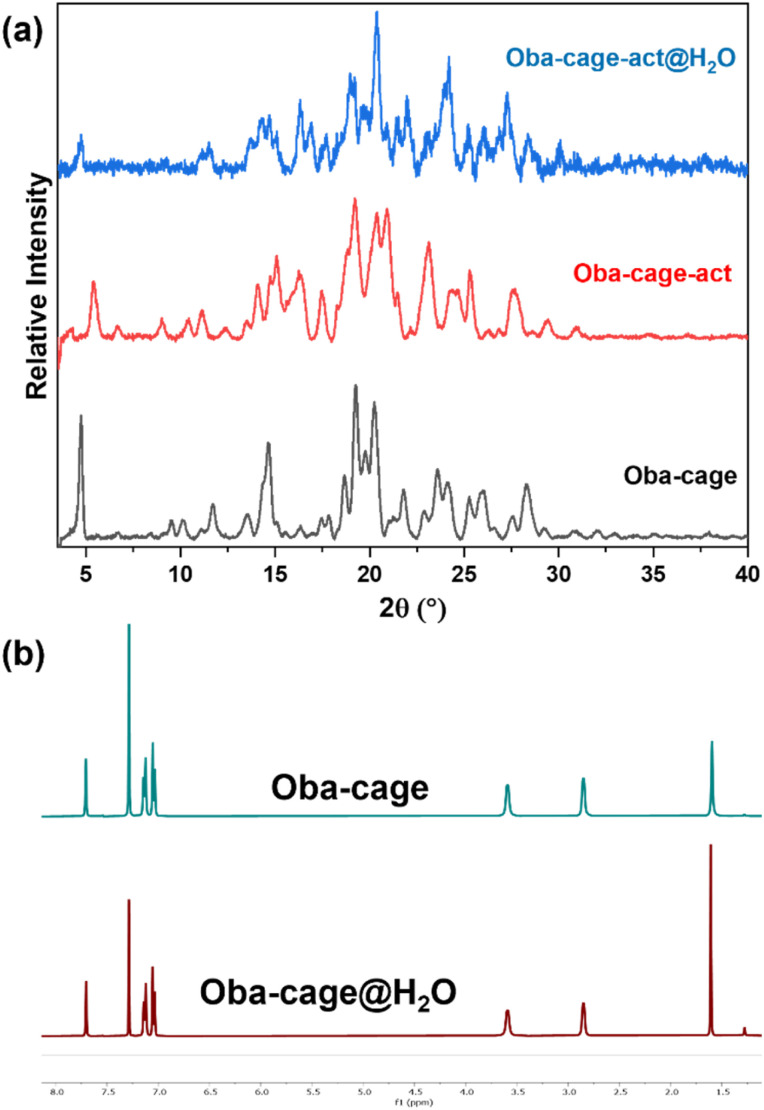
(a) PXRD patterns of the Oba-cage before and after water vapor adsorption measurements and (b) ^1^H-NMR spectra of the Oba-cage before and after exposure to water for four weeks.

Since the Oba-cage is not soluble in water due to the presence of hydrophobic frameworks which make growing crystals directly in water difficult, we believe it might also be difficult to form a complex with water molecules. To examine this and its stability further, the Oba-cage was exposed to water. The PXRD patterns, ^1^H-NMR spectra and TEM images of the Oba-cage before and after exposure to water for four weeks show that it is stable and not undergoing hydrolysis in water (Fig. 2, S9 & S10). However, when the Oba-cage crystallised from organic solvents such as THP in the presence of trace amounts of water, we obtained crystals with water molecules in their crystal structures as revealed by the SCXRD analysis. This observation prompted us to investigate the water adsorption performance of the Oba-cage further.

**Fig. 3:**
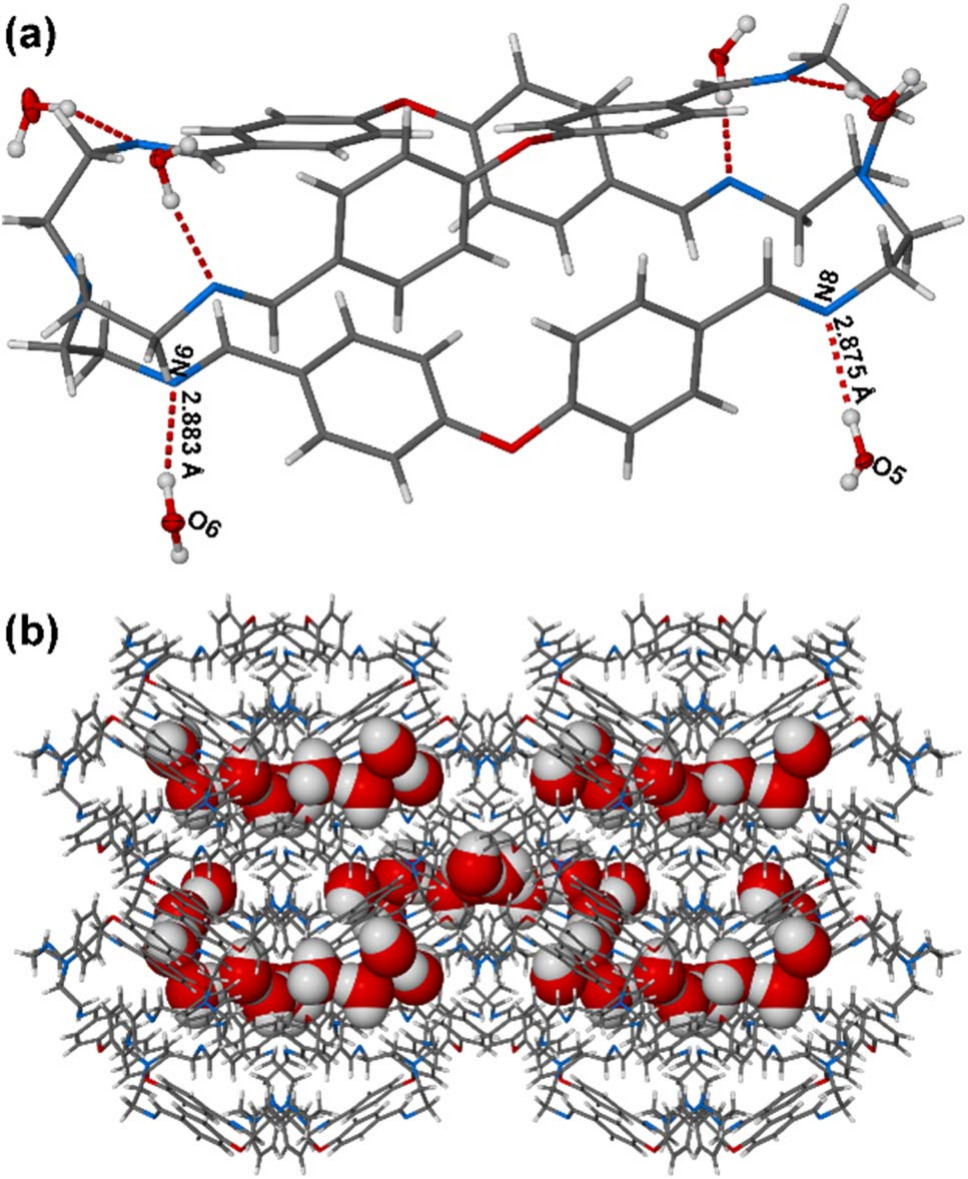
Perspective view showing (a) the hydrogen bonds between the Oba-cage and water and (b) water guest molecules (red) located in the channels of the crystal packing of Oba-cage@H_2_O.

The water uptake by the Oba-cage was confirmed by single crystal X-ray diffraction (SCXRD). From the single crystal structure analysis, approximately six molecules of water were found per Oba-cage molecule (Fig. 3 and Table S1). The six water molecules form strong host/guest O–H⋯N hydrogen bonds with the six imine nitrogen atoms of the cage forming a stable Oba-cage@H_2_O complex (Fig. S11 and Table S2). The water molecules occupied the extrinsic channel/void generated because of the inefficient packing of the Oba-cage which further confirms the nonporous nature of the Oba-cage (Fig. S12 & S13). We also observed a complete water network through guest/guest hydrogen bonding interactions confined in the extrinsic voids of the cage (Fig. S14 and Table S3). The formation of hydrogen bonds with water, despite the lack of solubility, suggests that water molecules can interact selectively with hydrophilic sites exposed on the crystal surface or within extrinsic voids created during crystallization.^17^ Such interactions may influence crystallization behaviour, guest uptake, or stability in humid environments. All these interactions support surface wettability and strong water storage/retention capacity displayed by the Oba-cage.

**Fig. 4:**
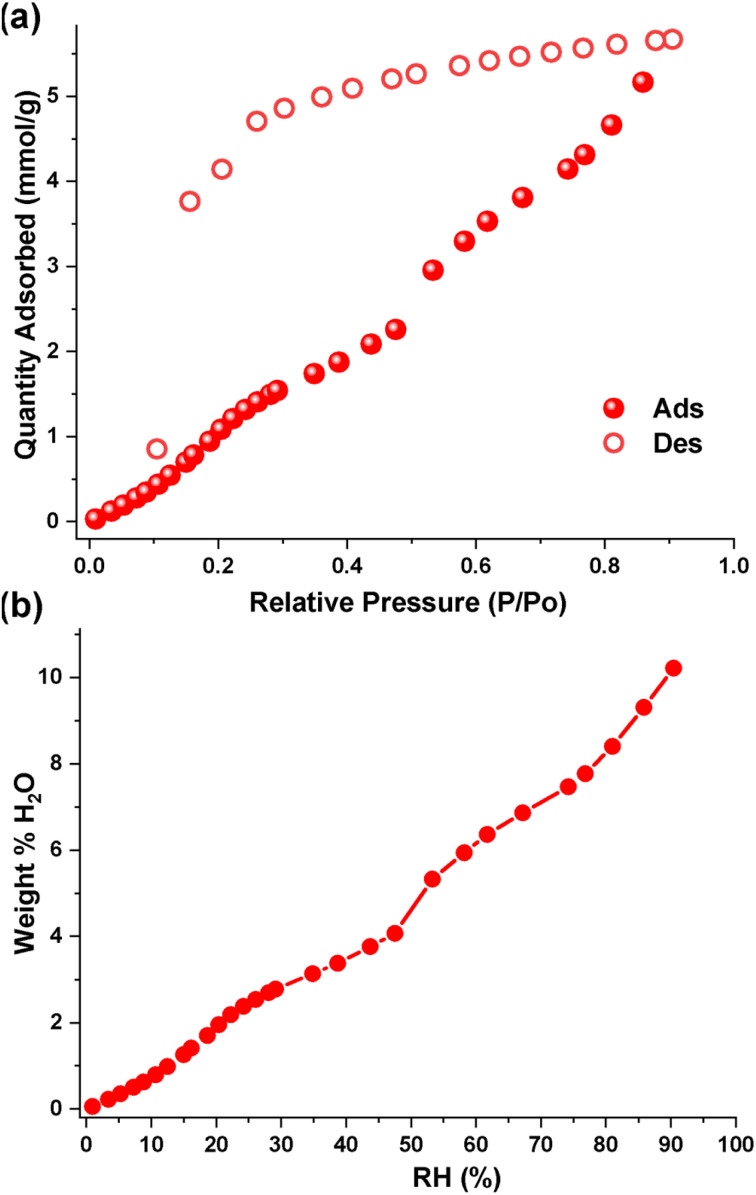
(a) Water vapor isotherm showing the quantity of water adsorbed by the Oba-cage at 25 °C and (b) the water uptake in wt% by the Oba-cage at different relative humidities.

To establish the water storage capacity of the Oba-cage further, we carried out water vapor sorption measurements at 25 °C using a 3Flex surface area analyzer. From the vapor sorption isotherm, the Oba-cage adsorbed 5.67 mmol g^−1^ of water vapor (Fig. 4). This amount of water adsorbed by the Oba-cage is quite significant owing to the nonporous nature of the Oba-cage itself. Furthermore, the large hysteresis observed between the adsorption and desorption isotherms in the water vapor sorption isotherm indicates that the Oba-cage has the capacity to store/hold water molecules under the condition of investigation (Fig. 4a). This observation is consistent with the revelations from the single crystal analysis. The Oba-cage shows a percentage water uptake of 10.2 wt% at the highest relative humidity of 90% (Fig. 4b). This uptake capacity at high relative humidity is unprecedented for nonporous crystalline organic cages.

To evaluate the water uptake and storage capacity of the Oba-cage under different practical temperature conditions, we further measured the water vapor adsorption capacity at 0 °C and 40 °C (Fig. S15). The water vapor isotherm at 0 °C shows a substantial water uptake with an adsorption capacity of 106 mg g^−1^ (5.90 mmol g^−1^) with persistent hysteresis which is slightly higher than 102 mg g^−1^ obtained at 25 °C. However, at a higher temperature of 40 °C, there is a significant decrease in the water uptake capacity of 37 mg g^−1^ (2.06 mmol g^−1^) (Fig. S15).^21^ The ability of the Oba-cage adsorbent to maintain a high-water capacity at 0 °C and 25 °C with a sharp decrease at 40 °C not only confirms its thermal sensitivity but also positions the cage as a suitable candidate for water harvesting and storage particularly in cool climates.^21,22^ The stability and reusability of the Oba-cage were confirmed through three consecutive water adsorption–desorption cycles with no significant loss in performance observed (Fig. S16).

Furthermore, the TGA result of the Oba-cage earlier exposed to water shows a weight loss of about 10% at around 100 °C (Fig. S17). This corresponds to the percentage of water adsorbed by the Oba-cage as revealed by the vapor sorption measurements. This result is therefore consistent with the vapor isotherm measurement result.

## Conclusions

In conclusion, we have successfully demonstrated the unprecedented high water uptake and storage capacity of a nonporous crystalline organic cage (Oba-cage). Although the Oba-cage is insoluble and stable in water without being hydrolyzed, it undergoes strong host/guest O–H⋯N intermolecular interactions with water molecules in the presence of a mixed organic solvent/water system as revealed by the crystal structure. This seemingly contradictory behaviour *i.e.* water interaction without solubility can be attributed to the presence of accessible polar functionalities (*e.g.*, imine nitrogen atoms) on the cage surface, which engage in localized hydrogen bonding with the surrounding water molecules. The water uptake capacities of 10.6 wt% and 10.2 wt% at 0 °C and 25 °C respectively are significant for a nonporous crystalline organic cage. This result, to the best of our knowledge, stands as the first example demonstrating such high water uptake and storage capacity by any nonporous crystalline organic cages. So, understanding such behaviour could prove valuable for applications in humidity sensing, guest encapsulation, or solid-state water storage.

## Author contributions

L. O. A. conceptualized and performed the major experiments and wrote the first draft. N. K., S K., W. L., and B. M. contributed to the characterization experiments. N. M. K. supervised the work and finalized the paper. All authors have given approval to the final version of the manuscript.

## Conflicts of interest

There are no conflicts to declare.

## Supplementary Material

SC-017-D5SC06328K-s001

SC-017-D5SC06328K-s002

## Data Availability

CCDC 2165792, 2165793 and 2469355 contain the supplementary crystallographic data for this paper.^23*a*–*c*^ All data are available in the submitted SI file. Supplementary information is available. See DOI: https://doi.org/10.1039/d5sc06328k.
