# Supplementary material for: A nonporous crystalline organic cage for selective water uptake and storage
Source: Chem Sci. 2025 Nov 10;17(1):511–5. doi: 10.1039/d5sc06328k (PMC12604641; doi:10.1039/d5sc06328k)
Supplement: SC-017-D5SC06328K-s001 [file SC-017-D5SC06328K-s001.pdf]

Supplementary Information (SI) for Chemical Science.
This journal is © The Royal Society of Chemistry 2025

A Nonporous Crystalline Organic Cage for Selective Water Uptake and Storage

Lukman O. Alimi,[a] Nida Khalfay,[a] Soumaya Khlifi,[a] Weibin Lin,[a] Basem Moosa[a] and Niveen M. Khashab*[a]

a Smart Hybrid Materials (SHMs) Laboratory, Advanced Membranes and Porous Materials Center, King Abdullah University of Science and Technology (KAUST), Thuwal 23955-6900, Kingdom of Saudi Arabia.

Acknowledgements

We thank King Abdullah University of Science and Technology (KAUST) for supporting this work.

Author Contributions

L.O.A conceptualized and performed the major experiments and wrote the first draft. N.K., S.K., W.L., and B.M contributed to the characterization experiments, reviewed and edited the manuscript. N. M. K. supervised the work and finalized the paper.

All authors have given approval to the final version of the manuscript.

Supporting Information

Materials and Methods

All reagents were commercially available and used as supplied without further purification. Compound **Oba-cage** was synthesized by modifying the previous literature report.[1]

Single Crystal Growth.

Single crystals of **Oba-cage@$CHCl_3$** were obtained by vapor diffusion of hexane into chloroform solution of **Oba-cage** at room temperature; Colourless single crystals of **Oba-cage@oCT** were also obtained after several days by vapor diffusion of o-Chlorotoluene into a THF solution of **Oba-cage** at room temperature. **Oba-cage@H_2O** crystals were obtained by slow evaporation of THP/water solution in 9.9:0.1 v/v ratio.

Single X-ray Crystal Structure Determination

Single crystal X-ray diffraction data were recorded on a Bruker D8 Venture equipped with a digital camera diffractometer using graphite-monochromated Mo Kα radiation (λ= 0.71073 Å) for the crystal structure. Data reductions were carried out by means of a standard procedure using the Bruker software package SaintPlus 6.01.[2] The absorption corrections and the correction of other systematic errors were performed using SADABS.[3] The structures were solved by direct methods using SHELXS-2008 and refined using SHELXL-2018.[4] X-Seed[5] was used as the graphical interface for the SHELX program suite. Data collection, structure refinement parameters and crystallographic data for the crystals are given in Table S1.

Activation of Oba-cage.

As-synthesized **Oba-cage** materials were activated under vacuum at 60 °C for 6 h to obtain the activated **Oba-cage**. While the activated **Oba-cage** after water adsorption can be regenerated upon heating at 100 °C under vacuum for 3 h.

Adsorption experiment using water vapor.

An open 5 mL vial containing 10 mg of activated **Oba-cage** was placed in a 20 mL vial containing 1 mL of water and left covered for four weeks.

NMR

NMR spectra were recorded on Bruker-400 (400 MHz for ^{1}H; 101 MHz for ^{13}C) instruments internally referenced to $SiMe_4$ signal.

PXRD

Routine powder X-ray diffraction (PXRD) data were collected using Cu Kα radiation (λ = 1.5418 Å, 40 kV and 30 mA) on a Bruker D8 ADVANCE instrument operating in Bragg-Brentano geometry. Intensity data were recorded using an X'Celerator detector, and 2θ scans in the range of 3.5 − 40° were performed with a step size of 0.02° at a scan speed of 0.02 (°/s). The sample was placed in a zero-background sample holder and normal configuration of the instrument was used.

TGA

Thermogravimetric analysis was carried out using an automatic sample loading TA Instruments 5500 analyzer. The samples were heated starting from room temperature to 700 °C using Nitrogen as protective gas.

BET Analysis

Low-pressure gas adsorption measurements were performed on a Micromeritics Accelerated Surface Area and Porosimetry System (ASAP) 2020 surface area analyzer. Samples were degassed under a dynamic vacuum for 12 h at 60 °C prior to each measurement. Nitrogen isotherms were measured using a liquid nitrogen bath (77 K).

Water Vapor Sorption and Humidity Analysis

Water vapor adsorption measurements were performed on a Micromeritics 3Flex-3500 surface area analyzer. Samples were degassed under a dynamic vacuum for 12 h at 60 °C prior to each measurement. Water vapor isotherms were measured using water bath at 273 K, 298 K and 313 K.

Synthesis of Oba-cage

Tris(2-aminoethyl)amine (Tren) (292.48 mg; 2.0 mmol) was dissolved in MeCN (5 mL) and was added dropwise over 1 h to the solution of 4,4'-Oxybis(benzaldehyde) (678.69 mg; 3.0 mmol) in MeCN (50 mL). The reaction mixture was stirred overnight at room temperature. A light-yellow precipitate was formed which was filtered and washed further with MeCN, then dissolved in dichloromethane and filtered to remove polymers. After the removal of dichloromethane, **Oba-cage** was obtained in 82% yield.

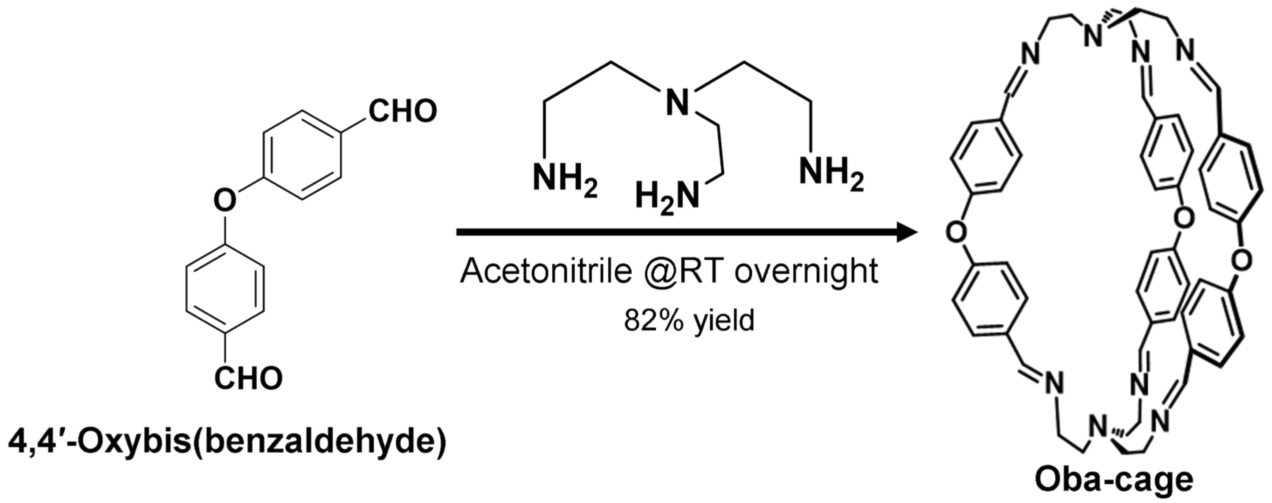

Scheme S1: Synthesis of 4,4’-Oxybis(benzaldehyde)-based imine Cage (Oba-cage).

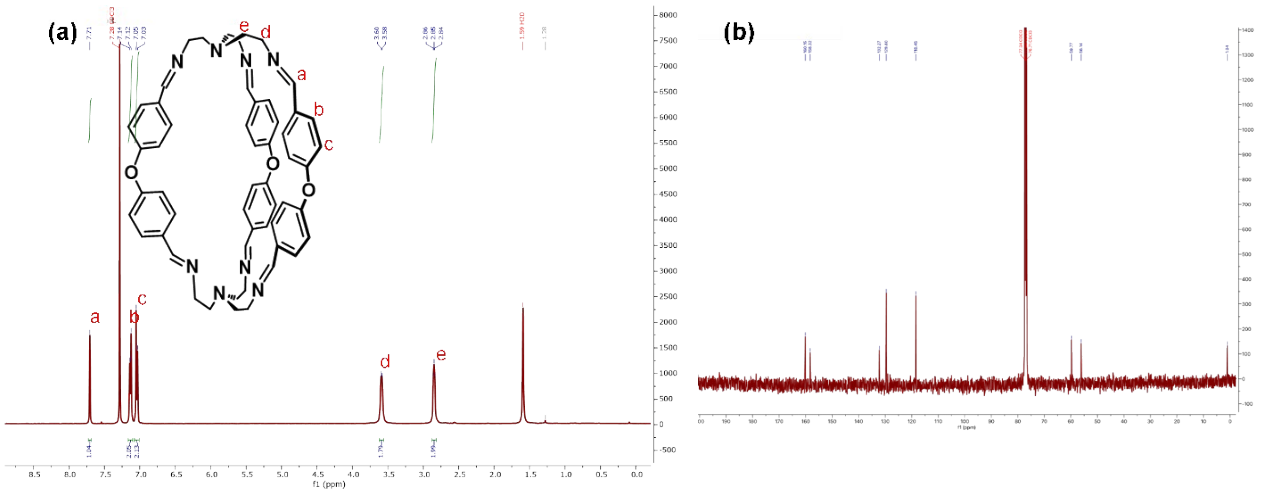

Figure S1: (a) H NMR spectrum (400 MHz, $CDCl_3$, 293 K) and (b) ^{13}C NMR spectrum of the Oba-cage.

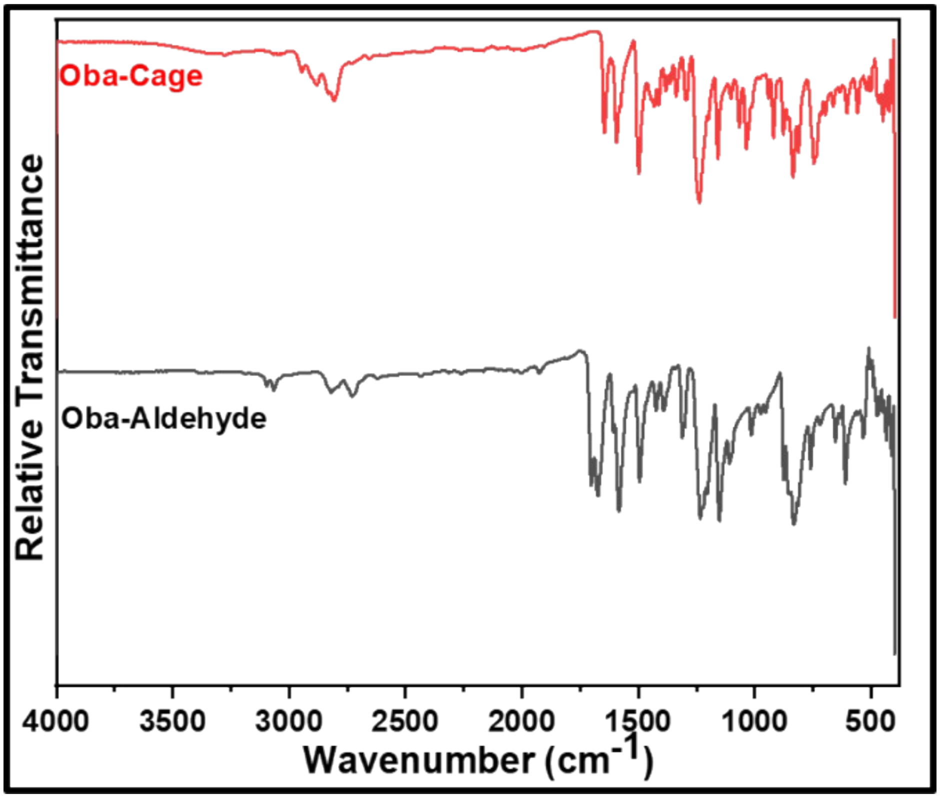

Figure S2: FT-IR spectra showing the formation of the Oba-cage.

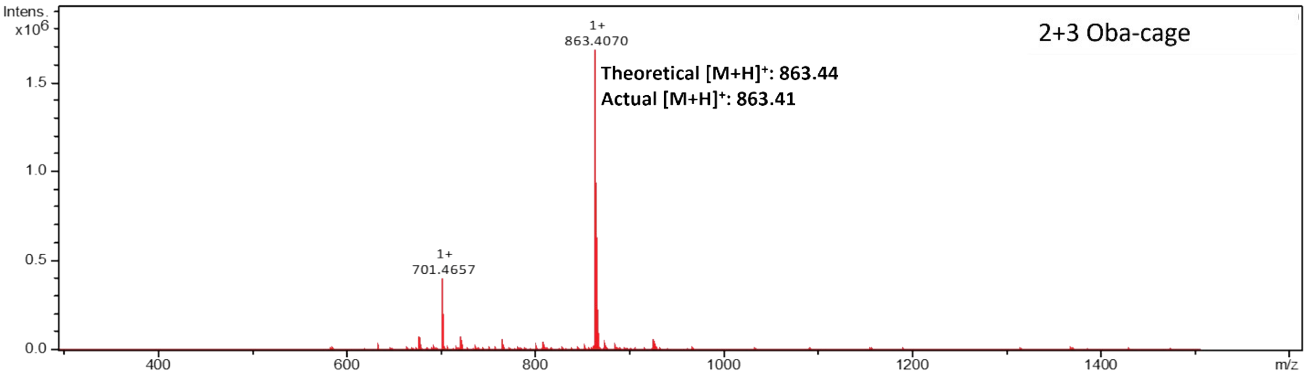

Figure S3: ESI-Mass spectrum of the Oba-cage.

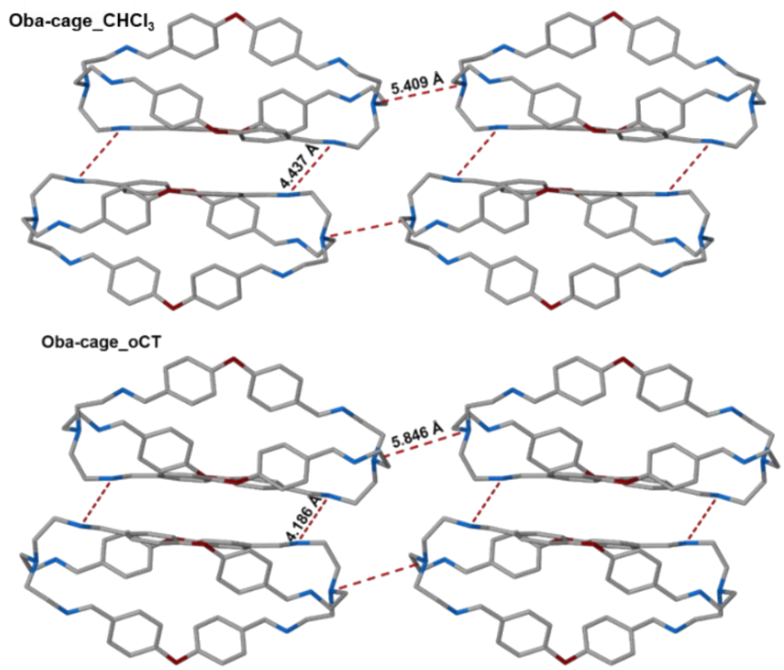

Figure S4: Perspective showing changes in the crystal packing of Oba-cage@$CHCl_3$ and Oba-cage@oCT.

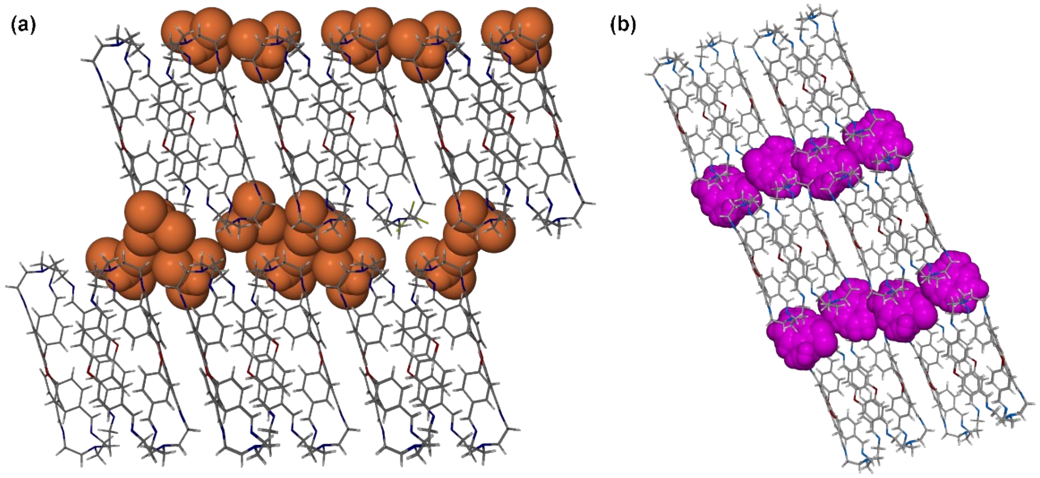

Figure S5: Crystal packing of (a) Oba-cage@$CHCl_3$ and (b) Oba-cage@oCT

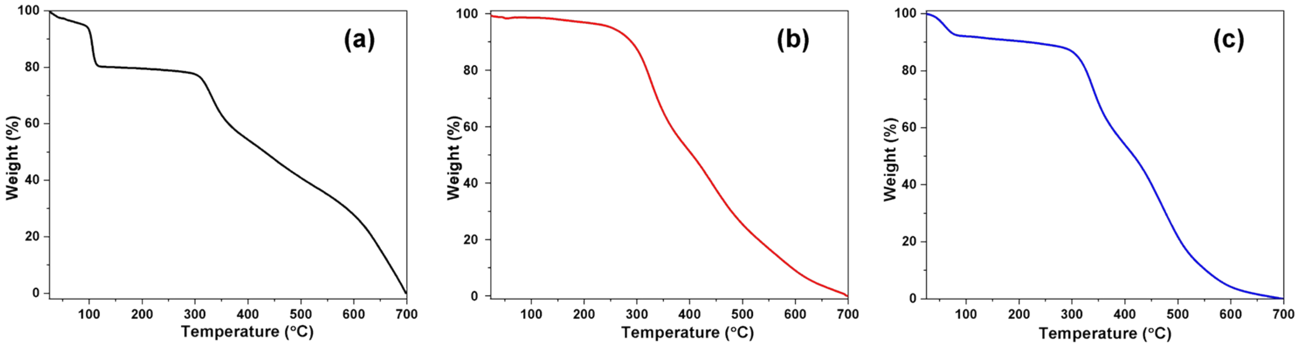

Figure S6: TGA curves of (a) as-synthesized, (b) fully activated and (c) water adsorbed Oba-cage.

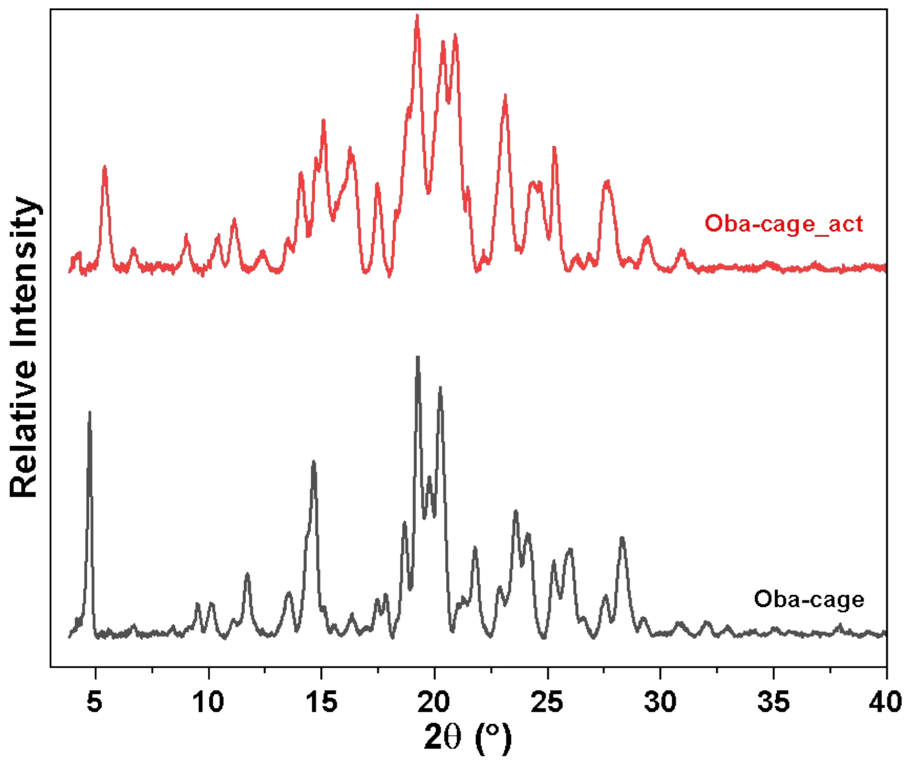

Figure S7: PXRD patterns of Oba-cage and fully activated Oba-cage.

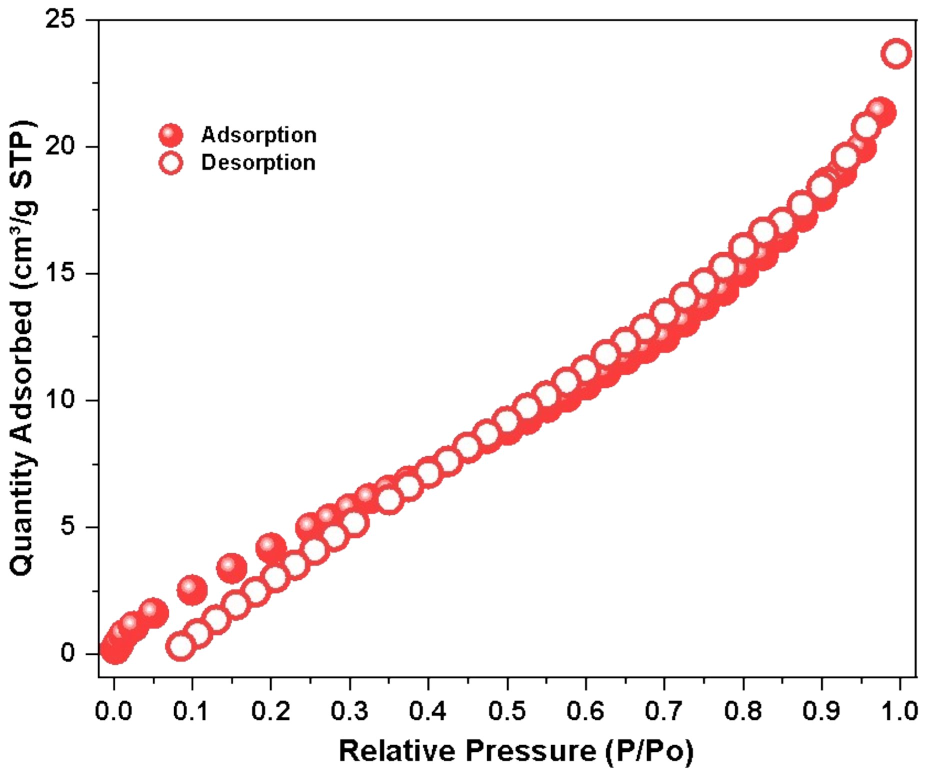

Figure S8: Nitrogen gas sorption isotherm (77K) of the activated Oba-cage.

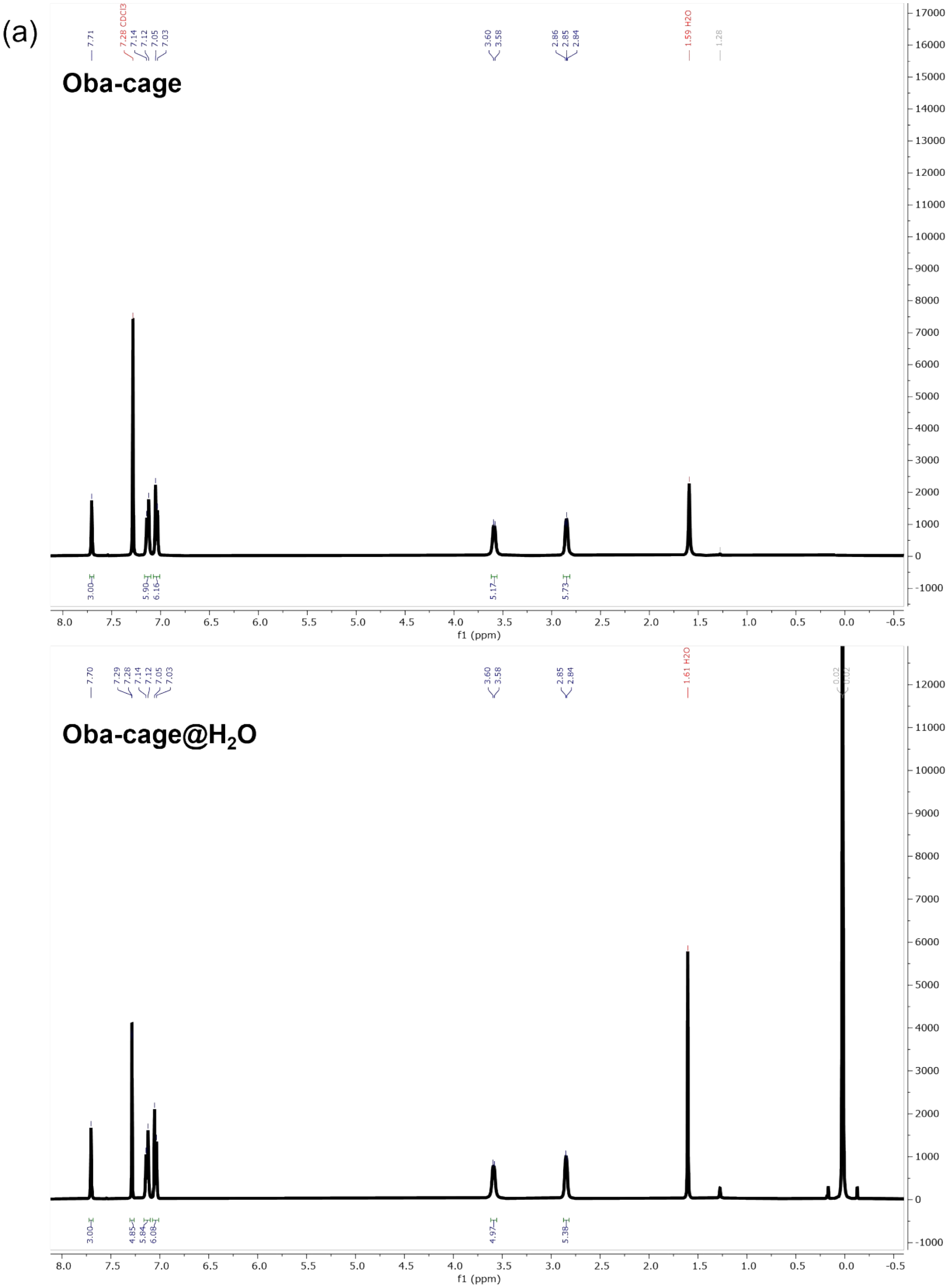

Figure S9a: H-NMR spectra of the Oba-cage before and after water adsorption experiments.

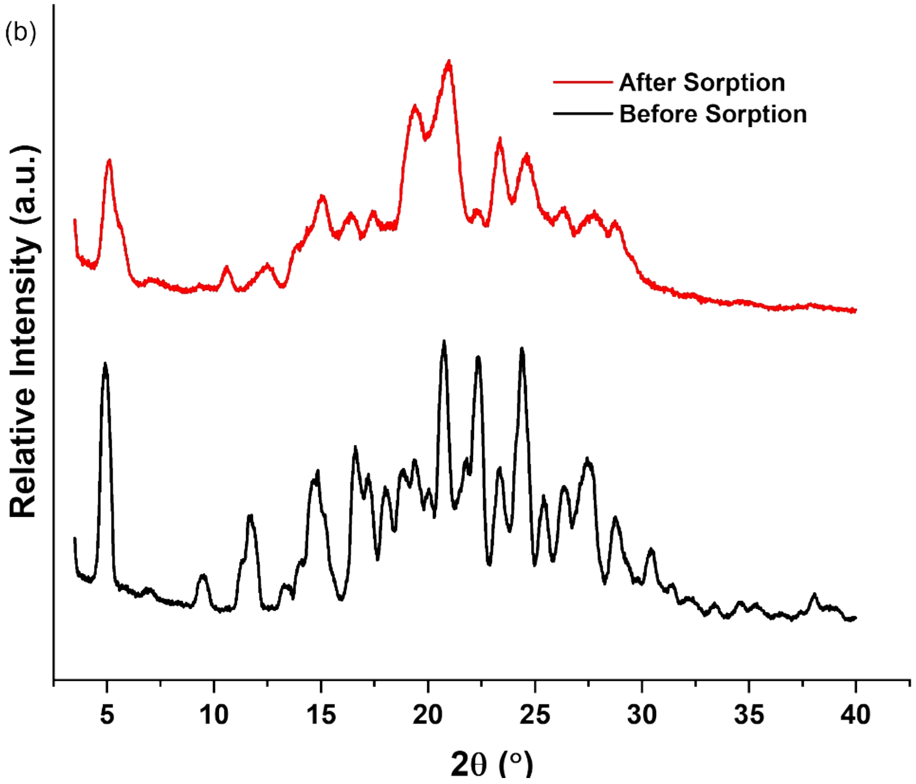

Figure S9b: PXRD patterns of the Oba-cage (a) before and (b) after the complete water adsorption/desorption cycle on the 3Flex analyzer.

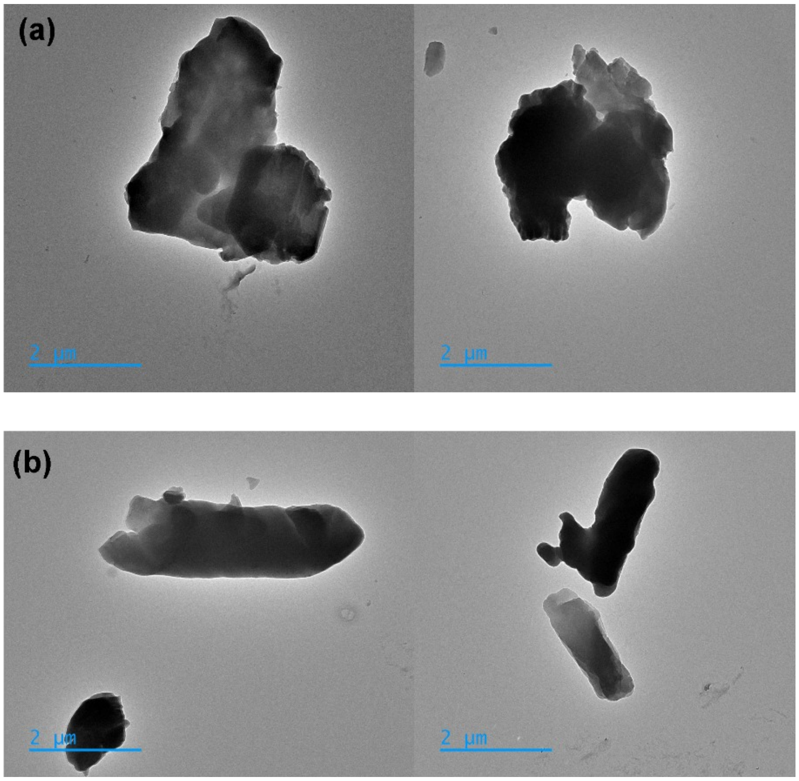

Figure S10: TEM images of the (a) Oba-cage and (b) Oba-cage after exposure to water for four weeks.

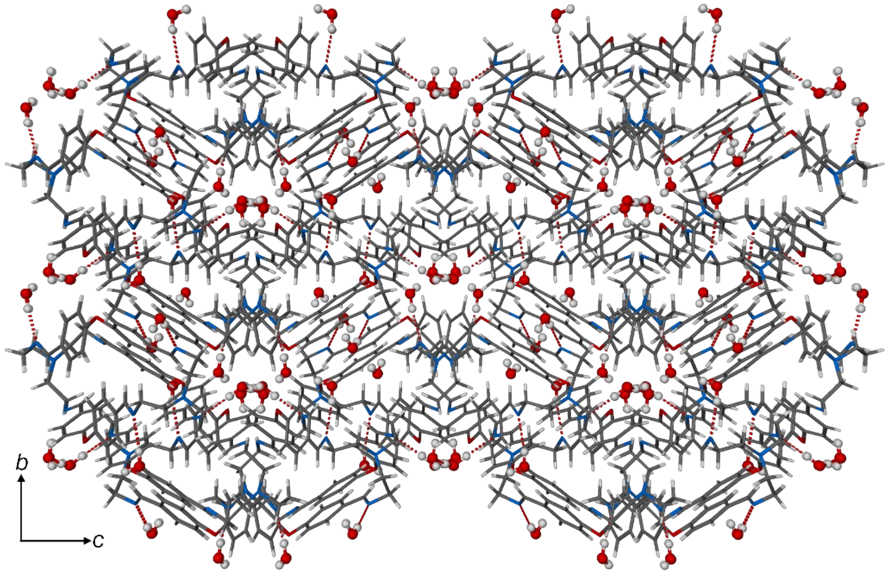

Figure S11: Perspective view showing the host/guest intermolecular hydrogen bonding interactions.

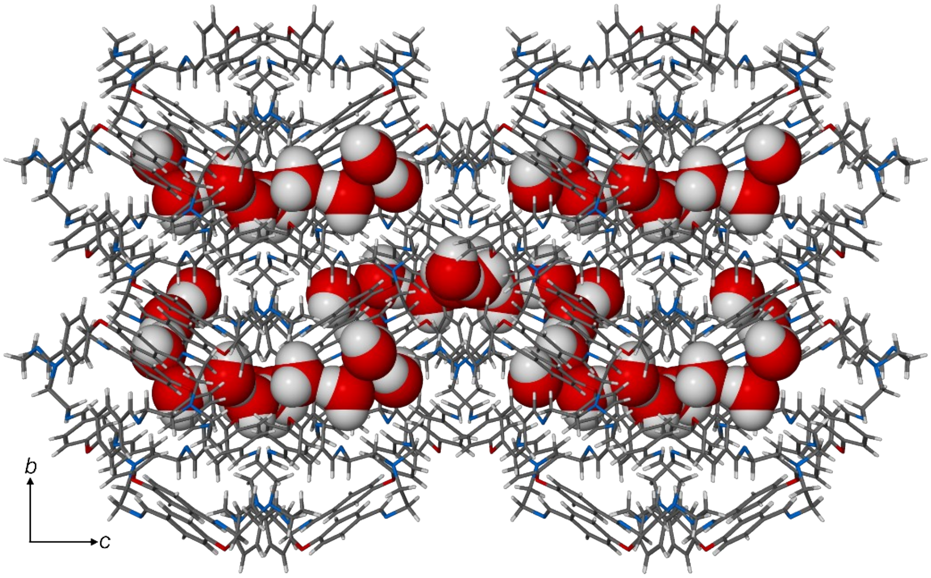

Figure S12: Perspective view showing the water guest molecules (space-fill model) in the extrinsic cavities of the Oba-cage.

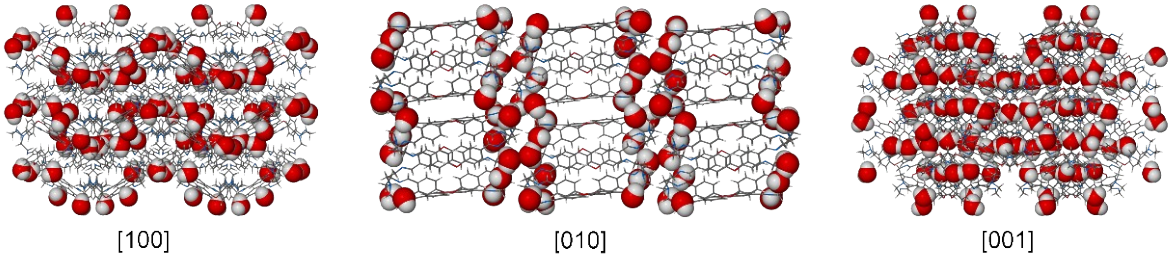

Figure S13: Crystal packing showing the water guest molecules (space-fill model) in the extrinsic cavities of the Oba-cage when viewed along all the crystallographic axes.

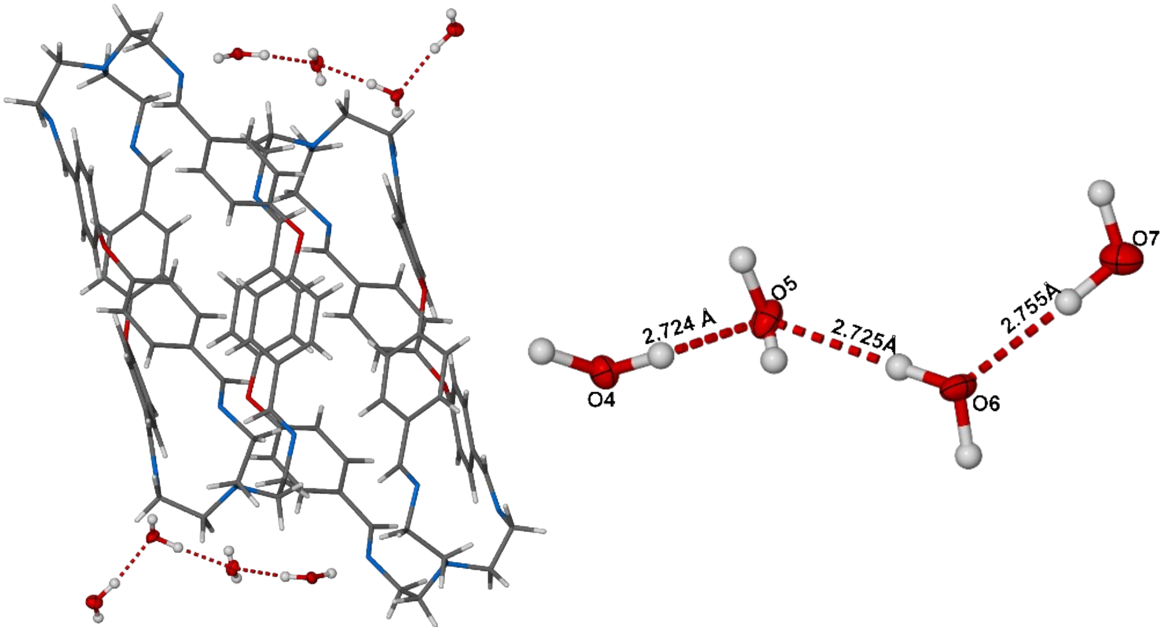

Figure S14: Crystal packing showing the water network through guest/guest hydrogen bonding interactions.

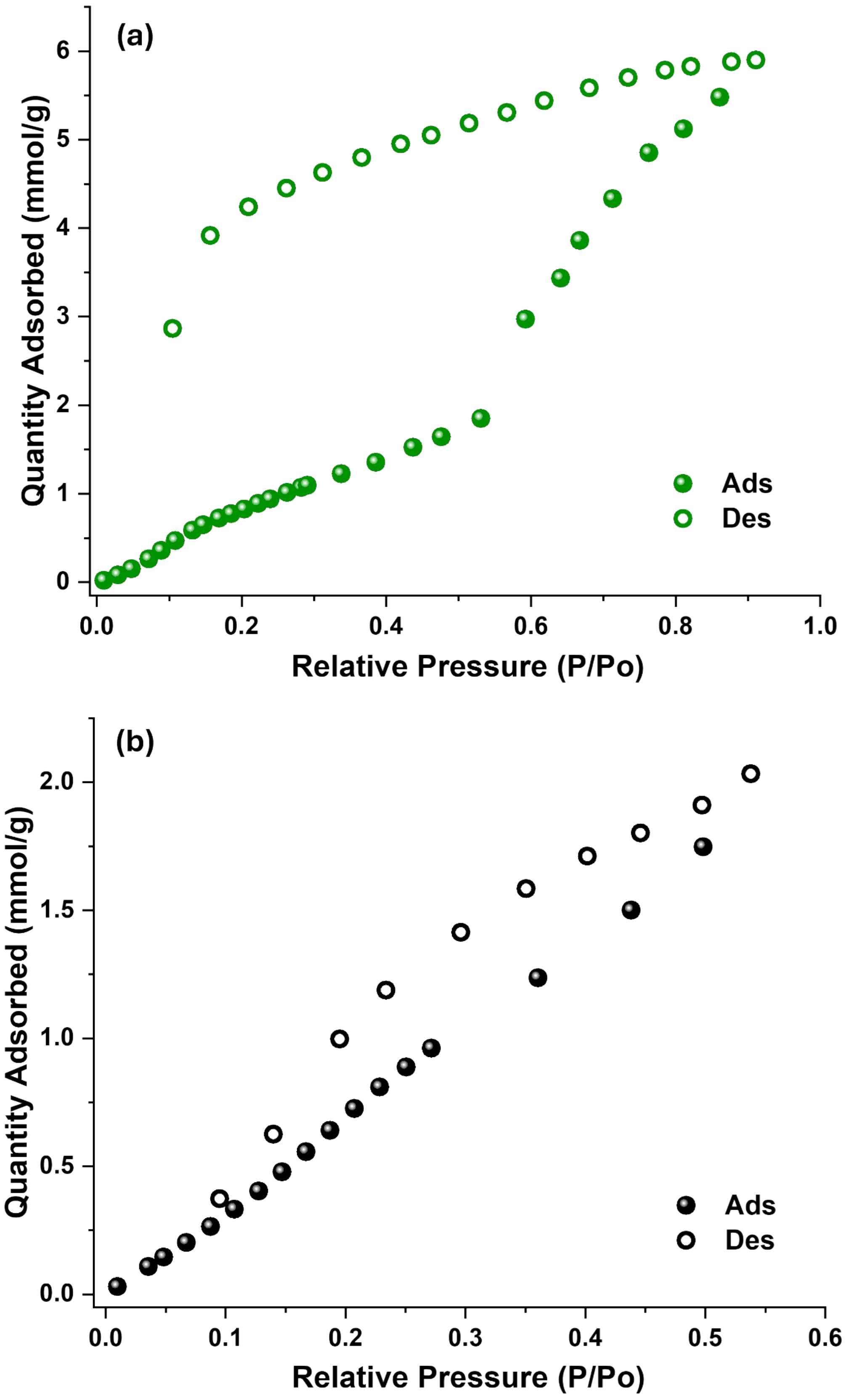

Figure S15: Water adsorption-desorption isotherm of the Oba-cage at (a) 0 °C and (b) 40 °C.

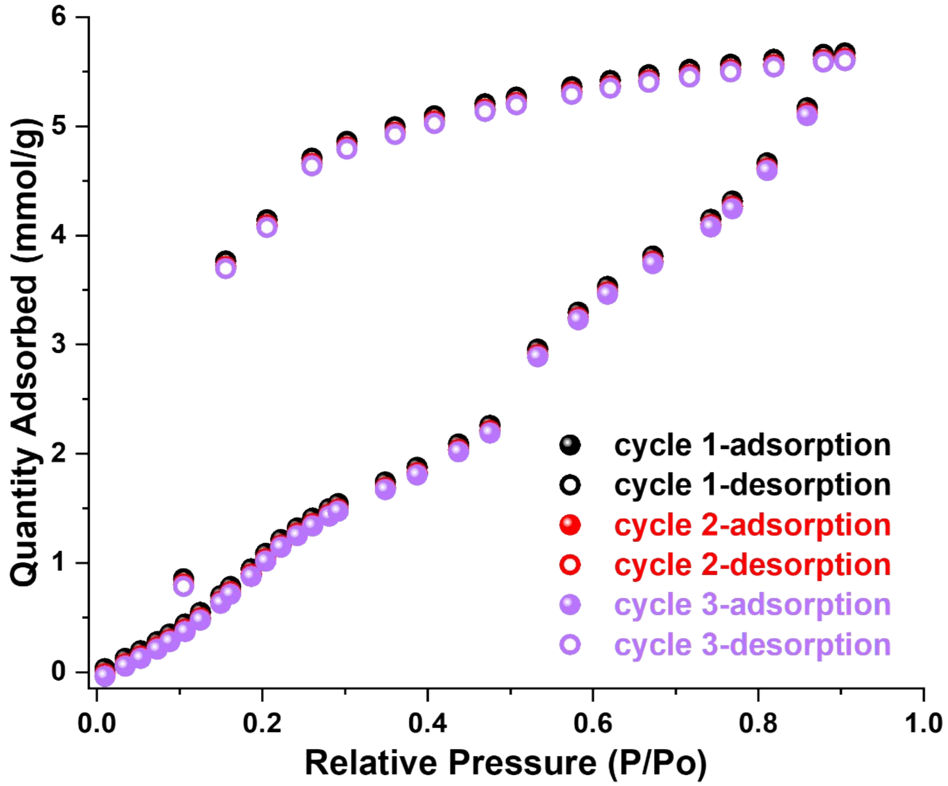

Figure S16: Water adsorption-desorption showing the stability and recyclability of the Oba-cage over three cycles.

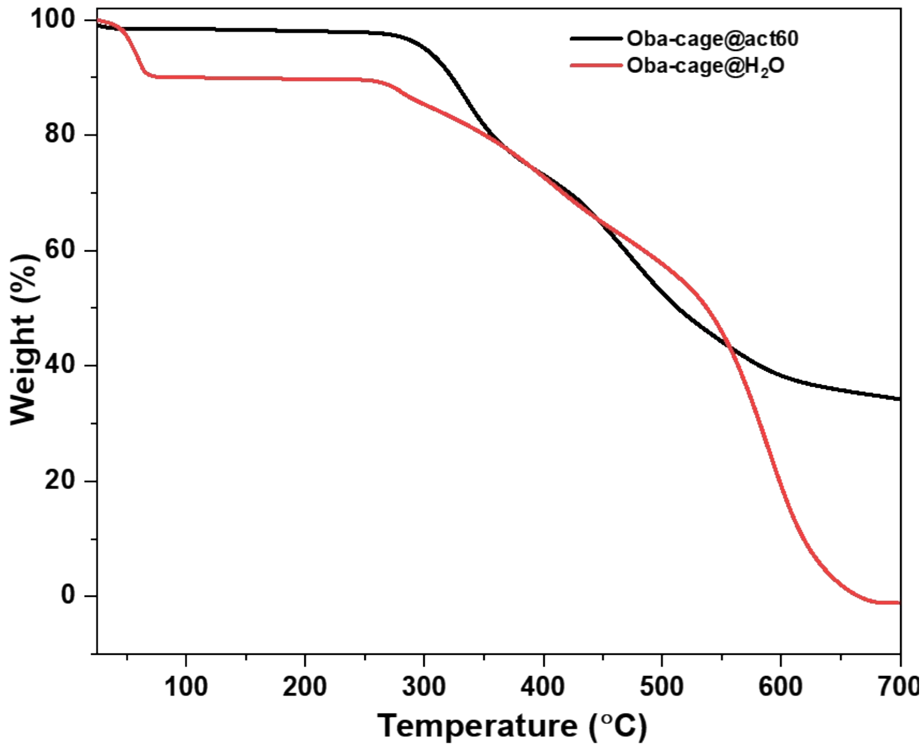

Figure S17: TGA curves showing the water uptake capacity of the Oba-cage.

Table S1: Crystallographic details of the Oba-cage in different solvents.

IDENTIFICATION CODE	Oba-cage@$CHCl_3$	Oba-cage@oCT	Oba-cage@H_2O
**Empirical formula**	$C_{56}H_{56}Cl_6N_8O_3$	$C_{61}H_{61}ClN_8O_3$	$C_{54}H_{66}N_8O_9$
**Formula weight (g/mol)**	1101	989.62	970.21
**Temperature /K**	120	120	120
**Crystal system**	Triclinic	Triclinic	Monoclinic
**Space group**	*P*-1	*P*-1	*P*2/*n*
***a*/ Å**	10.9208(9)	10.8687(4)	21.4759(12)
***b*/ Å**	13.5651(10)	12.8732(5)	10.6707(5)
***c*/ Å**	19.7896(15)	19.6339(8)	23.6099(13)
**α/°**	107.002(2)	77.2480(10)	90
**β/°**	94.441(3)	88.6220(10)	110.611(2)
**γ/°**	102.646(3)	79.1100(10)	90
**Volume/ $Å^3$**	2703.9(4)	2630.62(18)	5064.2(5)
**Z**	2	2	4
**ρcalcg/cm^3**	1.353	1.249	1.273
**F(000)**	1148	1048	2070
**Crystal size/mm^{-3}**	0.089 x 0.102 x 0.211	0.089 x 0.102 x 0.211	0.3 x 0.2 x 0.1
**Radiation**	MoKα (λ = 0.71073)	MoKα (λ = 0.71073)	MoKα (λ = 0.71073)
**reflections collected**	106286	58440	353130
**Independent reflections**	13364(R_{int} = 0.0282)	9219(R_{int} = 0.0517)	12571(R_{int} = 0.0472)
**Data/restraints/para meters**	11664/0/658	6309/348/732	10702/0/667
**Goodness-of-fit on F2**	1.032	1.092	1.068
**Final R indexes [I>=2σ (I)]**	*R*1 = 0.0399, *wR*2 = 0.0986	*R*1 = 0.0777, *wR*2 = 0.2215	R1 = 0.0504, *wR*2 = 0.1395
**CCDC**	**2165792**	**2165793**	**2469355**

Table S2: Some important host/guest hydrogen bonding intermolecular interactions between Oba-cage and water in Oba-cage@H_2O crystal structure.

Distance	D···A (Å)	H···A (Å)	>D–H···A (°)
**O4–H4B···N3**	2.918	2.019	171.08
**O5–H5A···N8**	2.875	2.028	164.63
**O6–H6B···N6**	2.883	2.016	173.35
**O7–H7A···N4**	3.004	2.150	166.57
**O8–H8B···N2**	2.897	2.055	162.38
**O9–H9B···N7**	2.987	2.153	160.47

Table S3: Some important guest/guest hydrogen bonding intermolecular interactions between in Oba-cage@H_2O crystal structure.

Distance	D···A (Å)	H···A (Å)	>D–H···A (°)
**O4–H4B···O5**	2.724	1.865	169.75
**O6–H5A···O5**	2.725	1.860	172.87
**O7–H6B···O6**	2.755	1.902	166.38

References

1. Kołodziejski, M.; Stefankiewicz, A. R.; Lehn, J. –M. Dynamic Polyimine Macrobicyclic Cryptands – Self-Sorting with Component Selection *Chem. Sci*., **2019**, 10, 1836–1843.

2. SAINT. Bruker AXS. Inc, Madison, Wisconsin, USA, 2014.

3. Sheldrick, G. M. SADABS. University of Gottingen, Germany, 2008.

4. Sheldrick, G. M. A Short History of SHELX. *Acta Crystallogr*. **2008**, A64, 112 -122.

5. Barbour, L. J. *Supramol. Chem*. **2001**, 1,189 –191.

6. Frisch, M. J. et al. (Wallingford, CT, 2016).
